# Rice Improvement Through Genome-Based Functional Analysis and Molecular Breeding in India

**DOI:** 10.1186/s12284-015-0073-2

**Published:** 2016-01-07

**Authors:** Pinky Agarwal, Swarup K. Parida, Saurabh Raghuvanshi, Sanjay Kapoor, Paramjit Khurana, Jitendra P. Khurana, Akhilesh K. Tyagi

**Affiliations:** National Institute of Plant Genome Research (NIPGR), Aruna Asaf Ali Marg, New Delhi, 110067 India; Interdisciplinary Centre for Plant Genomics and Department of Plant Molecular Biology, University of Delhi, South Campus, New Delhi, 110021 India

**Keywords:** Gene function, Genome, India, Marker-assisted selection, Rice, *Oryza sativa*, Transcriptomics

## Abstract

Rice is one of the main pillars of food security in India. Its improvement for higher yield in sustainable agriculture system is also vital to provide energy and nutritional needs of growing world population, expected to reach more than 9 billion by 2050. The high quality genome sequence of rice has provided a rich resource to mine information about diversity of genes and alleles which can contribute to improvement of useful agronomic traits. Defining the function of each gene and regulatory element of rice remains a challenge for the rice community in the coming years. Subsequent to participation in IRGSP, India has continued to contribute in the areas of diversity analysis, transcriptomics, functional genomics, marker development, QTL mapping and molecular breeding, through national and multi-national research programs. These efforts have helped generate resources for rice improvement, some of which have already been deployed to mitigate loss due to environmental stress and pathogens. With renewed efforts, Indian researchers are making new strides, along with the international scientific community, in both basic research and realization of its translational impact.

## Introduction

Rice feeds a large part of the world’s population, including Indians, and hence is an extremely important cereal crop. Sustained efforts have been made to improve its yield to meet the ever-increasing global demand. This has been possible largely through studies carried out on its agronomy, physiology, genetics and molecular biology. Since the genome sequence of an organism opens up new vistas to improve its performance, multiple efforts were simultaneously made to sequence *indica* and *japonica* subspecies of rice (*Oryza sativa*) genomes culminating in the availability of a map-based sequence of the genome from rice (International Rice Genome Sequencing Project 2005; reviewed in Vij et al. 2006). To this international initiative, India contributed by sequencing and assembly of the long arm of chromosome 11 and participation in mapping/annotation of the rice genome. This was achieved by the collaborative efforts of the researchers at the Department of Plant Molecular Biology, University of Delhi, South Campus, and National Research Center on Plant Biotechnology, Indian Council of Agricultural Research (Chen et al. 2002; International Rice Genome Sequencing Project 2005; The Rice Chromosomes 11 and 12 Sequencing Consortia 2005; Rice Annotation Project 2007, 2008). Recently, remarkable progress has been made in understanding genetic and functional diversity in rice by sequencing *Oryza glaberrima* (Wang et al. 2014) and 3000 other globally distributed accessions of rice (Li et al. 2014).

An annotated genome enlists the putative genes and their sequence catalogs. Subsequently, the encoded proteins by such genes can be classified into families depending on the presence of a conserved domain or motif. Once the locus IDs of genes are delineated, gene organization and structure can be analyzed, to determine unique features of genes and their products. Similarly, evolutionary and phylogenetic analyses help classify the family members into distinct classes and define their origin. Hence, the availability of the annotated rice genome has paved the way for the identification of members of multifarious gene families, India being a major contributor to this analysis. Many of these have been linked with the Rice Genome Annotation Project (RGAP) and are available at the community annotation project, while others are yet to be added (http://rice.plantbiology.msu.edu/annotation_community_families.shtml).

The next imperative step is to gain knowledge about the expression patterns of genes (Agarwal et al. 2014). For this, techniques like microarrays, RNA sequencing, microRNA sequencing and downstream analyses have been used to study transcriptomes, and their regulation by miRNA in multifarious developmental and stress conditions related to rice. Differentially regulated genes have been selected as targets for functional validation with the eventual aim to raise improved rice plants. This has been accomplished by generating transgenic plants with altered expression of the genes, followed by their detailed analysis. In this way, Indian researchers have been able to identify genes involved in development and stress response and elucidate downstream genes and pathways. For the larger benefit of the scientific community, the data generated has been deposited in international public repositories. To this effect, databases as well as bioinformatic tools are being created to facilitate easy access and seamless analysis of large data sets.

Both annotated genes and intergenic regions can be used to define functions of genes/quantitative trait loci (QTLs) by forward genetic approaches. Indian labs are making use of rich diversity of the rice genome for such purposes. The available genome/transcriptome sequences of diverse rice genotypes have been used to generate enormous resources in the form of genomic (genic) SSR (simple sequence repeat) and SNP (single nucleotide polymorphism) markers at a genome-wide scale especially in *indica* and aromatic rice (Parida et al. 2009; Jain et al. 2014). The large-scale validation and high-throughput genotyping of these genome/gene-based markers in diverse natural Indian germplasm (core and mini-core) collections, advanced generation mapping and mutant populations of rice are underway. These efforts assisted in mining of novel natural/functional allelic variants, understanding of molecular diversity and domestication pattern, construction of high-density genetic linkage maps and apply genetic/association mapping to identify potential genomic loci associated with complex quantitative traits of agronomic importance in rice (Sharma et al. 2005; Ngangkham et al. 2010; Marathi et al. 2012; Kumar et al. 2015). Marker-assisted selection (MAS) to introgress and pyramid superior functional genes (alleles)/QTLs regulating complex yield/quality component and stress tolerance traits into diverse Indian rice genotypes for their genetic enhancement has been attempted (Singh et al. 2011a). In this article, an attempt has been made to highlight some key investigations from India in the post-genomic era that help gain knowledge about molecular biology and genetics along with its applications to improve the rice crop. Several of these findings were also presented during the 11th International Symposium on Rice Functional Genomics held in India in 2013.

## Review

### Loci for 3394 Genes from 50 Families Annotated

Transcription factors (TFs) are integral components of any regulatory and/or metabolic pathway. Comprehensive genome wide analyses of their gene structure and expression have revealed interesting aspects about their regulation during development and stress and provided leads for their functional characterization. Amongst the first detailed analysis of large TF gene families, the C_2_H_2_ zinc-finger family, encoded by 189 *ZOS* genes was found to have 97 new members, including 10 previously unnannotated members. Also, new types of zinc fingers were discovered, apart from the cannonical ones. These genes were found to differentially express during reproductive development and abiotic stresses (Agarwal et al. 2007). The rice genome was found to encode for 75 MADS box TFs. Nine members belonging to the Mβ group of the MADS-family, which was considered absent in monocots, were also identified (Arora et al. 2007). Eighteen new bZIP motif-encoding genes were identified, making the total to 89 genes for this class of TFs. Sixty-two genes out of 72 intron-containing genes had the intron within the bZIP region (Nijhawan et al. 2008). The analysis of homeobox gene family showed that HD-ZIP III sub family was intron-rich, resulting in a high number of alternatively spliced genes. Also, 50 % of the genes had evolved due to duplication events (Jain et al. 2008c). Most of the HSFs were found to be induced by heat (Mittal et al. 2009), apart from other developmental and stress conditions. Splicing of a member resulted in the exclusion of its NES (Chauhan et al. 2011). Many DOF genes were found to be expressed during various stages of seed development (Gaur et al. 2011) and it is known that DOFs play an important role in the process (Agarwal et al. 2011). The promoters of some of the *MYB* genes were found to have novel *cis* elements and lower arms of chromosomes had relatively more *MYB* genes (Katiyar et al. 2012). Thus, a comprehensive analysis of members of each TF family brings to light certain unique features, which remain unnoticed during whole genome annotation.

Besides TFs, a number of gene families coding for various signal transduction components (STCs) have also been characterized for clues to their structure-function relationships. Amongst hormone signaling pathways, 31 *OsAux/IAA* genes were identified and most of them were found to be up-regulated upon auxin treatment, six of them at much higher levels (Jain et al. 2006a). Another auxin responsive family, *GH3*, encoding amido synthetases involved in catalyzing the synthesis of IAA-amino acid conjugates, was found to have 12 members in rice. Group III genes of this family are apparently absent not only in rice but other monocots too (Jain et al. 2006b). The early auxin responsive intronless gene family *SAUR* has 58 members, 17 of which are clustered on chromosome 9 (Jain et al. 2006d). Kinases and phosphatases are essential parts of any signaling pathway. Rice genome has been found to code for 31 CDPKs, two of which were newly identified (Ray et al. 2007). The identification protocol led to 192 and 70 previously unidentified genes as coding for RLCKs (Vij et al. 2008) and MAPKKKs, respectively (Rao et al. 2010). Most of the 11 SERK/SERLs had been previously annotated incorrectly and were found to express in tissues other than somatic embryos as well (Singla et al. 2009). Also, 173 members for three classes of LecRLKs were identified (Vaid et al. 2012). For ten CBLs, 33 interacting CIPKs were found. Many of these interactions were found to be conserved from *Arabidopsis* (Kanwar et al. 2014). Five families of phosphatases were found to have been encoded by 132 genes (Singh et al. 2010a). Also, 51 genes were found to code for 73 TCS proteins, including 14 histidine kinases, 5 phosphotransfer proteins and 32 response regulator encoding genes. They were found to be clustered at various regions on rice chromosomes (Pareek et al. 2006). Amongst other STCs, 687 F-box protein encoding genes were identified in rice, many of which had other domains as well (Jain et al. 2007). Another class of STCs comprises of known stress inducible genes. Rice genome was found to have 18 abiotic stress inducible *SAP* genes (Vij and Tyagi 2006), heat stress and other abiotic stress inducible 23 sHsps (Sarkar et al. 2009) and only five *Hsp100* genes (Batra et al. 2007). J-proteins are co-chaperones to Hsp70 and rice has 104 such genes, coding for proteins with high molecular weights. Five such genes could even rescue the corresponding yeast mutant under heat stress (Sarkar et al. 2013). Amongst transporters, rice genome was found to code for 33 calcium transporters, which included one channel, 14/15 ATPases and 16 exchangers (Goel et al. 2011; Kamrul Huda et al. 2013), 133 ABC transporters (Saha et al. 2015) and 14 sulphate transporters. The sulphate transporters are expressed during sulphate starvation, heavy metal and abiotic stress as well as reproductive development (Kumar et al. 2011). Apart from these, phospholipase A and C components have also been identified from rice genome (Singh et al. 2013a; Singh et al. 2012a).

The identification of genes encoding transcriptional regulators in rice has its share of contribution from India. In effect, eight, 19 and five genes coding for Dicer-like proteins, Argonautes and RNA-dependent RNA polymerases have been identified (Kapoor et al. 2008). Apart from this, 115 RNA helicases and 31 DNA helicases have also been identified (Umate et al. 2010). Rice genome has been found to code for 51 Mediator complex protein coding genes, which include all subunits identified so far in various organisms (Mathur et al. 2011). Eleven regulators belonging to various families have the KIX domain, which is responsible for protein-protein interactions (Thakur et al. 2013). Gene family analysis often brings out interesting aspects of the genome. A pair of chymotrypsin protease inhibitor encoding genes, out of a total of 17, shares a bidirectional promoter (Singh et al. 2009). Also, genes coding for an assortment of other proteins have been identified. This includes 48 genes coding for the enzyme glutaredoxin (Garg et al. 2010), 11 for peroxiredoxins (Umate 2010), 14 for lipoxygenase (Umate 2011; Marla and Singh 2012), 11 for Class I metallothioneins induced under heavy metal stress (Gautam et al. 2012), 28 for cyclophilins (Trivedi et al. 2012), 14 for glyoxalases I and II (Mustafiz et al. 2011), 16 carotenoid biosynthesis genes (Chaudhary et al. 2010) and 158 *ARMADILLO* genes, which have an ARM repeat (Sharma et al. 2014a). Fifty-nine CBS domain containing proteins have also been found to contain other domains, and hence may perform diverse functions (Kushwaha et al. 2009). Thus, in totality, locus IDs have been assigned to the members of several important gene families along with insights into their expression profiles during development and abiotic stress conditions. Also, the interacting partners and/or promoter sequences have been analyzed for a few members. The general points of interest include (i) possibility of identifying new genes and clades for a family on the basis of molecular model-based comparisons, (ii) conservation in position of introns, (iii) compensating evolutionary selection of mutations in protein structure (iv) identification of state-specific or inducible genes of a family and (v) identification of gene functions that have evolved specifically in monocots.

### Databases and Tools are Being Used for Storing and Mining Information

As more information is getting accumulated in plant biology, databases are being created to make the information publically available, for easy access and analysis. These databases have a user friendly interface for easy data retrieval (Agarwal et al. 2014). Indian rice scientists have developed various databases on varied aspects. For QTLs related to abiotic stress, QlicRice provides not only the genomic locations, but also the responsible locus IDs (Smita et al. 2011). STIFDB2 documents all stress responsive TFs in both *indica* and *japonica* rice. Apart from an extensive *cis* regulatory element analysis, it can be used for network prediction as well (Naika et al. 2013). Similarly, RiceSRTFDB has records of stress-responsive TFs along with their expression patterns, mutants and *cis* element analysis (Priya and Jain 2013).

A new concept for literature based ‘Manually Curated Database of Rice Proteins’ has been developed based on novel data curation methods that enable digitization and semantic integration of experimental data. The current release of database has data of 2401 rice proteins manually curated from 538 research articles. Over 800 phenotypic/biochemical traits have been curated along with their associated genes (Gour et al. 2014). The availability of the genome sequence allows researchers to perform multiple analysis, such as elucidation of intronless genes (Jain et al. 2008a) and prediction of miRNA targets (Archak and Nagaraju 2007). Various tools have been developed and validated using rice genome sequence, such as RetroPred for prediction of non-LTR retrotransposons (Naik et al. 2008), a machine learning tool to predict infection by rice blast fungus depending on the weather conditions (Kaundal et al. 2006), MirtronPred to predict plant mirtrons (Joshi et al. 2012) and pTAREF for prediction of miRNA targets (Jha and Shankar 2011). The URLs for all the webservers and softwares have been mentioned in Table 1.

**Table 1:** Details of databases and web tools developed by Indian rice scientists

Sl. no.	Name of database/ tool	URL	Information generated	Reference
1	Rice blast infection prediction	http://www.imtech.res.in/raghava/rbpred/	Predicts chances of rice blast infection by using weather conditions as an input.	Kaundal et al. 2006
2	RetroPred	http://www.juit.ac.in/attachments/RetroPred/home.html	A downloadable tool which detects repeat regions in genomic sequences and classifies them as LINEs and SINEs.	Naik et al. 2008
3	pTAREF	http://scbb.ihbt.res.in/new/p-taref/form1.html	A downloadable tool to predict miRNA targets in transcriptome data.	Jha and Shankar 2011
4	QlicRice	http://nabg.iasri.res.in:8080/qlic-rice/	A database of 974 abiotic stress-related QTLs connected with 460 locus IDs, with their physical and genetic data, which can be easily queried.	Smita et al. 2011
5	MirtronPred	http://203.92.44.117/mirtronPred/prediction.php	Uses intronic sequences as an input to predict mirtrons.	Joshi et al. 2012
6	STIFDB2	http://caps.ncbs.res.in/stifdb2/	Encompasses over 5000 stress responsive genes from Arabidopsis and both *indica* and *japonica* rice, with a list of putative TF binding sites in their promoters.	Naika et al. 2013
7	RiceSRTFDB	http://www.nipgr.res.in/RiceSRTFDB.html	Salt and drought stress-responsive TFs from 99 Affymetrix chips can be queried.	Priya and Jain 2013
8	MCDRP	http://www.genomeindia.org/biocuration/	Manually curated data from all published rice genes, including ontology, functions, pathways and interactions.	Gour et al. 2014

### Transcriptome Analysis Presents a Snapshot of Participating Genes

Prior to the advent of microarray and RNA seq, other strategies including subtractive hybridization were used to determine the transcript levels of multiple genes (Agarwal et al. 2014). After the sequences of rice BAC and PAC clones were available, genes were identified from drought-stressed ESTs, some of which were novel (Babu et al. 2002; Gorantla et al. 2007; Reddy et al. 2002). In order to identify genes responsible for salt tolerance, subtractive cDNA libraries have been compared between tolerant (Pokkali) and susceptible (IR64) genotypes (Kumari et al. 2009). The samples that have undergone transcriptome analysis till date fall into various categories. Multiple developmental stages, tissues subjected to a/biotic or nutrient stress, developmental stages under stress, and resistant genotypes have been used to elucidate various genes/pathways operating in the desired situation. We have performed microarray analysis on 19 stages of rice development, including both vegetative and reproductive phases, and the data have been deposited at the Gene Expression Omnibus of NCBI. Along with this, microarray data from three stages of abiotic stresses, namely, cold, dehydration and salt, have also been deposited (Agarwal et al. 2007; Arora et al. 2007; Ray et al. 2011; Sharma et al. 2012). Also, the transcriptome dynamics during entire anther development from pre-pollen mother cell stage to mature anthers with tri-nucleate pollen have been analyzed by using microarrays to reveal genes specifically expressing during meiosis and playing potential roles in sporophyte to gametophyte switching during male gametophyte development (Deveshwar et al. 2011). Thidiazuron treated rice callus has also been analyzed by microarray to identify genes related to differentiation (Chakrabarty et al. 2009). The gene expression analysis of rice roots subjected to heavy metal stress helped in the identification of genes that can be modulated for detoxification (Dubey et al. 2010; Dubey et al. 2014). In different studies, microarray analyses have been performed on root, leaf and panicle under drought stress (Smita et al. 2013) and on two contrasting genotypes under drought stress (Lenka et al. 2011), rice samples subjected to heat and/or oxidative stresses (Mittal et al. 2012a, 2012b) and heat stress followed by recovery (Sarkar et al. 2014). With respect to biotic stress, microarray has been done for *Xanthomonas* infected rice plants (Grewal et al. 2012). Amongst nutrient stress, microarray analysis of calcium and phosphate starved plants shows involvement of various biosynthetic pathway genes such as carbohydrate, phosphate, lipid and nitrogen metabolism (Shankar et al. 2013; Shankar et al. 2014). Thus, whole genome transcriptome analyses have been done for diverse abiotic and biotic stress situations and various developmental stages in rice and they have helped in delineating essential genes and the associated pathways. These high quality transcriptome analyses have added to the efforts of the international community of rice researchers in generating an indispensible resource for future studies.

### Applications of Genome Sequencing for Functional and Regulatory Aspects

There are many scientific groups in India working on understanding regulation of various aspects of rice development and stress responses; they have been able to pin point the specific function of important genes by raising and analyzing transgenic plants with modified expression patterns. *RFL*, a rice ortholog of *LEAFY* from Arabidopsis, controls flowering time and architecture (Deshpande et al. 2015; Rao et al. 2008; Table 2). Further, transcriptome analysis of rice plants with altered expression of a desired gene by overexpression or silencing or mutation, along with phenotypic analysis, identifies the functional relevance of the gene. The downstream genes and networks can also be elucidated by such an analysis (Agarwal et al. 2014). A mutant *ewst1* in Nagina22 results in enhanced water tolerance and genes related to production of osmoprotectants and secondary metabolites are up regulated in it (Lima et al. 2015). Ectopic expression of *OsiSAP1* or *OsSAP11* or *OsRLCK53* results in up regulation of genes imparting drought tolerance (Giri et al. 2011; Dansana et al. 2014; Mukhopadhyay et al. 2004). Plants overexpressing *OsMKK6* have also been analyzed by microarray, indicating the role of this gene in stress regulation (Kumar and Sinha 2014). Microarray analysis of plants with overexpression and RNAi constructs of *OsMADS29* showed that the gene controls cytokinin mediated starch biosynthesis during seed development (Nayar et al. 2013). Nuclear translocation of this TF has been shown to be regulated by way of homodimerization and interaction with at least 19 other seed-expressing MADS-box proteins (Nayar et al. 2014). The gene *Pi54* in Taipei309 imparts tolerance to *Magnaporthea* infection by activating defense responsive pathways (Gupta et al. 2012). Thus, downstream operable genes and pathways have been elucidated. This has given valuable information on various genes and their related pathways. Table 2 enlists rice genes for which transgenic plants have been made in a homologous system.

**Table 2:** Some rice genes and promoters analyzed to elucidate their activities in transgenic rice

Sl. no.	Gene	Locus ID^a^	Construct	Function	Reference
1	*ANS* (Anthocyanidin synthase)	LOC_Os01g27490	OE	Increase in flavonoids and anthocyanins and hence oxidation potential	Reddy et al. (2007)
2	*ETHE1* (*ETHYLMALONIC ENCEPHALOPATHY PROTEIN 1*)	LOC_Os01g47690	promoter	Stress and calcium responsive	Kaur et al. (2014)
3	*GLYOXALASEII*	LOC_Os09g34100	OE	Confers salt stress tolerance	Singla-Pareek et al. (2008)
4	*IPK1* (inositol 1,3,4,5,6-pentakisphosphate 2-kinase)	LOC_Os04g56580	RNAi	Silencing of *IPK1*decreases the antinutrient phytic acid	Ali et al. (2013a)
5	*MIPS* (myo-inositol-3-phosphate synthase)	LOC_Os03g09250	RNAi	Decrease in phytate and increase in inorganic phosphate and iron levels	Ali et al. (2013b)
6	*NBS-Str1* (nucleotide binding site-LRR) *and BLEC-Str8* (β-lectin domain protein)	LOC_Os02g09790 and LOC_Os04g01950	Promoter	Cause increased expression under stress	Ray et al. (2012)
7	*orfB*	LOC_Os12g34018	RNAi	Unedited mitochondrial *orfB* gene causes male sterility by decreasing ATP synthase activity	Chakraborty et al. (2015)
8	*OsbHLH*, *OsFbox, OsiPK*	LOC_Os01g18870, LOC_Os05g46050, LOC_Os12g12860	Promoter	Responsible for anther-specific expression	Khurana et al. (2013)
9	*OsCDR1* (aspartate protease)	LOC_Os01g08330	OE	Provides resistance against fungal and bacterial pathogens	Prasad et al. (2009)
10	*OsClpB-cyt* (ClpB-cytoplasmic /Hsp100)	LOC_Os05g44340	Promoter	Classical heat shock element supresses expression under unstressed conditions	Singh et al. (2012b)
11	*OsCPK31* (CDPK)	AK110341^b^	OE, RNAi	Helps in rice grain filling	Manimaran et al. (2015)
12	*OsDREB2A* (Dehydration-responsive element binding transcription factor)	LOC_Os01g07120	OE	Osmotic, dehydration and salt stress tolerance	Mallikarjuna et al. (2011)
13	*OsGLP1* (germin-like protein)	LOC_Os08g35760	RNAi	Controls plant height and disease resistance	Banerjee and Maiti (2010)
14	*OsGLYII-2* (*GLYOXALASEII*)	LOC_Os03g21460	OE	Provides salinity and decarbonyl stress tolerance	Ghosh et al. (2014)
15	*OsGRX8* (glutaredoxin)	LOC_Os02g30850	RNAi	Abiotic stress response	Sharma et al. (2013)
16	*OSIAGP* (Arabinogalactan)	LOC_Os06g21410	Promoter	Provides pollen-preferential expression	Anand and Tyagi (2010)
17	*OsiPA*	LOC_Os10g40090	Promoter	Confers pollen-specific expression	Swapna et al. (2011)
18	*OsiSAP1*	LOC_Os09g31200	OE	Positively regulates water deficit stress by affecting gene expression leading to loss of membrane damage and lipid peroxidation	Dansana et al. (2014)
19	*OsiSAP8*	LOC_Os06g41010	OE	Abiotic stress tolerance	Kanneganti and Gupta (2008)
20	*OsiWAK1* (wall associated kinase)	LOC_Os11g46860	RNAi	Plant growth and development	Kanneganti and Gupta (2011)
21	*OsMADS1*	LOC_Os03g11614	OE	Involved in panicle, lemma and palea development	Prasad et al. (2001)
22	*OsMADS29*	LOC_Os02g07430	RNAi, OE, Protein-protein interaction	Regulates starch biosynthesis during seeds development by regulating active cytokinin levels	Nayar et al. (2013); Nayar et al. (2014)
22	*OsMKK6*	LOC_Os01g32660	OE	Participates in salt stress signaling and regulates phytoalexin biosynthesis due to UV exposure	Kumar and Sinha (2013); Wankhede et al. (2013)
23	*Osoxo4* (oxalate oxidase)	LOC_Os03g48780	OE	Resistance to sheath blight	Molla et al. (2013)
24	*OsSAG12-1* (senescence associated gene)	LOC_Os01g67980	RNAi	Negatively regulates cell death	Singh et al. (2013d)
25	*OsSUV3* (*SUPPRESSOR OF VAR3*, DNA and RNA helicase, ATPase)	LOC_Os03g53500	OE, RNAi	Provides tolerance against salt stress by upregulation of multiple hormones, resistance against metal stress	Sahoo et al. (2014a); Sahoo et al. (2014b); Tuteja et al. (2013)
26	*Pi54*	LOC_Os11g42010	OE	Provides resistance to *Magnaporthea* irrespective of insertion position	Arora et al. (2015)
27	*Rab16A*, 4X ABRE, 2X ABRC	LOC_Os11g26790	Promoter	Responsible for ABA responsive expression under stress and development	Ganguly et al. (2011)
28	*RFL* (*RICE FLORICULA/ LEAFY*)	LOC_Os04g51000	promoter and intron	Introns have regulatory regions	Prasad et al. (2003)
29	*RFL* (*RICE FLORICULA/ LEAFY*)	LOC_Os04g51000	RNAi, OE	Controls flowering, promotes axillary meristem initiation and hence tillering through auxin and strigolactone signaling	Deshpande et al. (2015); Rao et al. (2008)
30	*Ubi1* (Ubiquitin) from rice	LOC_Os06g46770	Promoter	Confers high levels of constitutive expression	Bhattacharyya et al. (2012)

^a^based on RGAP version 7 (http://rice.plantbiology.msu.edu/)

^b^represents cDNA clone from KOME (http://cdna01.dna.affrc.go.jp/cgi-bin/sogo.cgi?pj=598&class=598&page=cDNA) as no RGAP locus ID has been found

Many genes of rice have been characterized by their altered expression in a heterologous system as Arabidopsis and tobacco. Although these model plants are adopted for the ease with which the phenotype can be easily scored, in most cases, the results can be extrapolated to rice. For example, not only rice plants overexpressing OsDREB2A are abiotic stress tolerant (Mallikarjuna et al. 2011), the gene OsDREB1B confers similar phenotype in tobacco as well (Gutha and Reddy 2008). Even in the case of promoters, rice sucrose synthase1, *RSs1* promoter confers phloem-specific expression in Arabidopsis (Saha et al. 2007). Such promoter sequences can be used for targeted gene expression. Ectopic expression of many abiotic stress responsive rice genes in Arabidopsis and/or tobacco makes plants tolerant to abiotic stress responses. Prominent amongst these are stress associated proteins, SAP1/11, receptor-like cytoplasmic kinase, OsRLCK253 (Giri et al. 2011), topoisomerases OsTOP6A1, OsTOP6A3 and OsTOP6B (Jain et al. 2006c, 2008b), OsTCP19 (Mukhopadhyay and Tyagi 2015), a bZIP TF in the Saltol QTL, OsHBP1b (Lakra et al. 2015), cyclin, OsCyp2-P (Kumari et al. 2014), protein phosphatase 2C, OsPP108 (Singh et al. 2015a), glutathione S-transferases, OsGSTU4 and OsGSTL2 (Sharma et al. 2014b; Kumar et al. 2013), glutaredoxin, OsGRX8 (Sharma et al. 2013), cystathionine β-synthase, OsCBSX4 (Singh et al. 2012c), Ca(2+)ATPase, OSACA6 (Kamrul Huda et al. 2014; Huda et al. 2013), metallothionein, OsMT1e-P (Kumar et al. 2012), myoinositol phosphate synthase from wild rice, PcINO1 (Patra et al. 2010; Das-Chatterjee et al. 2006; Majee et al. 2004), LEA protein, Rab16A (RoyChoudhury et al. 2007) and chymotrypsin protease inhibitor, OCPI2 (Tiwari et al. 2015b). On the biotic stress front, the promoter of *CYP76M7* is *Magnaporthea* inducible and can be used for expression of defense related genes (Vijayan et al. 2015). *OsSAP1* enhances tolerance to pathogen infection (Tyagi et al. 2014). Proteins with multiple roles have also been characterized in heterologous systems. Multidrug and toxic compound extrusion proteins, OsMATE1 and 2 have roles in both abiotic and biotic stresses and development (Tiwari et al. 2014). Germin-like protein1, OsGLP1, provides tolerance to both abiotic and biotic stress (Banerjee and Maiti 2010; Banerjee et al. 2010).

Likewise, functions of few miRNAs have also been elucidated. Since miRNAs cause degradation of their target mRNAs, the two express inversely (Raghuram et al. 2014). Osa-MIR414, osa-MIR164e and osa-MIR408 have been found to regulate the expression of *OsABP*, *OsDBH* and *OsDSHCT*. These miRNAs are down regulated during salinity stress (Macovei and Tuteja 2012). Thus, during salt stress, miRNAs targeting DEAD box helicases are down regulated (Macovei and Tuteja 2012; Umate and Tuteja 2010). Micro RNAs are also being identified by sequencing, such as those involved in tungro virus infection and salt stress (Sanan-Mishra et al. 2009). Drought tolerant genotype, Nagina 22, shows a variety of miRNAs differentially expressed during ‘anthesis’ stage drought, which may be responsible for its tolerance (Kansal et al. 2015). Further, miR408 which targets several plantacyanin genes is regulated differentially in Nagina 22 as compared to drought sensitive rice variety during drought stress (Mutum et al. 2013). Another study on salt-responsive miRNA markers has highlighted the differences in miRNAs amongst a sensitive and tolerant genotype of rice (Mondal and Ganie 2014). Moreover, over half of the miRNA targets have been found to be conserved amongst *indica* and *japonica* genotypes (Archak and Nagaraju 2007). miRNAs are also responsible for low N-tolerant genotypes (Nischal et al. 2012). The antagonistic effects of arsenic and selenium are controlled by the miRNA population (Pandey et al. 2015). Also, varieties showing variance in tolerance to arsenite stress show differences in miRNA accumulation (Sharma et al. 2015). Eleven TFs, controlled by miRNAs, regulate abiotic stress responsive genes (Nigam et al. 2015). Non-conserved miRNAs have also been predicted in rice (Kumar et al. 2014). Rice has been found to have polycistronic *MIR166*s (Barik et al. 2014). Submergence responsive miRNA targets (Paul and Chakraborty 2013) and TF binding motifs in miRNAs (Devi et al. 2013) have been predicted. miRNA species are differentially and preferentially expressed amongst various developmental stages (Mittal et al. 2013) and callus differentiation (Chakrabarty et al. 2010). They are even responsible for the cross talk amongst biotic and abiotic stresses (Sanan-Mishra et al. 2009). The studies discussed above provide evidence how the annotation of the rice genome has been useful in determining the gene networks and also unraveling the function of genes involved in various aspects of development in rice and when it is exposed in unfavorable conditions.

### Genome-Wide Development and Use of Informative Genetic Markers

The availability of gold standard reference genome sequence of *japonica* rice cv. Nipponbare (International Rice Genome Sequencing Project 2005) has propelled the genome resequencing and transcriptome sequencing of diverse rice genotypes in recent years by use of NGS (next-generation sequencing) approaches in India. This in turn led to the development of enormous resources in the form of genomic (genic) simple sequence repeat (SSR) and single nucleotide polymorphism (SNP) markers at a genome-wide scale in rice. Despite availability of 2240 RM (rice microsatellite) and 18,828 hyper-variable class I SSR markers from rice draft and/or whole genome sequences (McCouch et al. 2002; International Rice Genome Sequencing Project 2005), many initiatives have been undertaken by Indian researchers to develop multiple kinds of novel concept-based informative genomic and genic SSR markers at a genome-wide scale for expediting high-throughput genetic analysis in rice. Preliminary efforts have been made to utilize 33,722 rice unigene sequences (48.8 Mb) for developing a novel class of non-redundant 13,230 genic microsatellite markers designated as UniGene derived MicroSatellite (UGMS) markers, which are bin-mapped across 12 rice chromosomes (Parida et al. 2006). Considering the added advantages of these markers in assaying allelic variation in transcribed genic sequence components of the genome, these have been placed in public domain as a web-based freely accessible relational database, *U*g*M*icro*S*at*db* (*U*nigene *M*icro*S*atellite database) for unrestricted use (Aishwarya and Sharma 2007). In another study, 17,966 novel “GNMS (genic non-coding microsatellite)” markers targeting different upstream regulatory (5′UTRs and promoters) and non-coding (3′UTRs and intronic) sequence components of protein coding genes annotated from Nipponbare rice genome were developed, which are bin-mapped across 12 rice chromosomes (Parida et al. 2009). This further led to the identification of 112 orthologous and paralogous “CNMS (Conserved Non-coding MicroSatellite)” markers from the putative rice promoter sequences for comparative genome mapping, and understanding of evolutionary and gene regulatory complexities among rice and other members of grass family (Parida et al. 2009). The GNMS and CNMS markers with their many desirable genetic attributes, including high polymorphic potential and functional significance have efficiency to serve as candidate gene-based microsatellite markers in diverse genomics-assisted breeding applications in rice. Subsequently, utilizing the complete rice genome sequence information, 436 well-validated HvSSR (highly variable SSR) markers with repeat-length of 51–70 bp have been developed at a genome-wide scale (Singh et al. 2010b). The efficacy of these markers for exhibiting consistent PCR amplification and detecting higher allelic polymorphism among accessions even by a cost-effective agarose gel, have demonstrated their suitability in large-scale genotyping applications in rice. In order to develop trait-specific genetic markers, salinity-responsive microRNAs (miRNAs) have been targeted to develop 130 miRNA-SSR markers for their efficient utilization in salinity stress tolerance marker-assisted genetic enhancement of rice (Mondal and Ganie 2014). The genetic variation and evolutionary dynamics between *indica* cv. 93–11 and *japonica* cv. Nipponbare have been understood by identifying and analysing the repeat-units mutability of ~50,000 *in silico* polymorphic SSR markers occurring in diverse coding and non-coding sequence components of rice genome (Grover et al. 2007).

In addition to SSR markers, the whole genome resequences/pseudomolecules and transcriptomic sequences of multiple rice genotypes enabled rapid discovery and development of genomic and genic SNP and InDel (insertion-deletion) markers *in silico* at a genome-wide scale. Recently, an international initiative, i.e., “The 3000 rice genome sequence project” has been completed to generate 20 million SNPs by genome resequencing of 3000 rice genotypes (Alexandrov et al. 2015). Similar efforts also have been made by Indian scientists to generate genome/gene-derived SNP and InDel markers by low-coverage resequencing specifically of diverse *indica* genotypes, including aromatic rice. For instance, non-redundant 2495052 SNP and 324034 InDel markers have been discovered by comparing the NGS-based whole-genome resequencing data of six elite *indica* inbred lines (three of each cytoplasmic male sterile and restorer lines) to accelerate genomics-assisted breeding for hybrid performance in rice (Subbaiyan et al. 2012). Subsequently, the whole genome resequencing of three drought/salinity tolerant (Nagina 22 and Pokkali) and sensitive (IR64) rice accessions identified non-redundant 1784583 SNPs and 154275 InDels between reference Nipponbare and three resequenced rice accessions. Based on this outcome, genome-wide 401683 SNPs between IR64 and Pokkali and 662509 SNPs between IR64 and Nagina 22 that are well-distributed across coding and non-coding regions of these sequenced genomes were discovered with the eventual aim to deploy them in marker-assisted breeding for abiotic stress tolerance in rice (Jain et al. 2014). More recently, the comparison of whole genome resequencing data of a widely cultivated low glycemic index-containing *indica* rice variety, Swarna, with reference genome Nipponbare, identified 1,149,698 SNPs (65,984 non-synonymous SNPs) and 104,163 InDels for deciphering the genetic basis of complex glycemic index quantitative trait in rice (Rathinasabapathi et al. 2015).

Essentially, the genomic and genic SSR and SNP markers developed especially by Indian initiatives are amenable to large-scale validation and high-throughput genotyping at the whole genome level. These informative markers are thus suitable for multi-dimensional genomics-assisted breeding applications.

### Characterization of Core/Mini-Core Germplasm and Mutant Resource for Rice Genetic Enhancement

Rice is grown in the wide range of agro-ecological conditions and adapted to diverse sociocultural traditions prevalent in India. Thus, Indian rice germplasm lines are rich in trait diversity. The rice germplasm resources, including cultivated varieties, breeding lines, landraces, wild accessions representing diverse agro-climatic regions of India as well as the world, have been conserved efficiently in different National germplasm repository centers like National Bureau of Plant Genetic Resources (NBPGR, New Delhi). The phenotypic and molecular characterization of the huge Indian rice germplasm collections is, therefore, vital in order to use the natural allelic diversity information in genomics-assisted rice crop improvement program. Considering the economic importance of aromatic rice in Basmati trade and commerce, significant efforts have been made for varietal identification and understanding the genetic diversity and domestication patterns majorly among different traditional (landraces) and evolved (elite) long- and short-grained Basmati and between Basmati and non-Basmati *indica* rice accessions (Nagaraju et al. 2002; Aggarwal et al. 2002; Sajib et al. 2012; Meti et al. 2013; Vhora et al. 2013). For example, the distinctness, uniformity and stability (DUS) in an aromatic rice variety, Pusa Basmati1, has been established by its precise phenotyping for diverse aroma and grain quality traits and large-scale genotyping of genome-wide SSR markers (Singh et al. 2004). The efficiency of genome-wide SSR markers and five mitochondrial gene-specific CAPS (cleaved amplified polymorphic sequences) markers for testing the genetic purity of Pusa6A parental lines during seed production of Pusa Rice Hybrid10 has been demonstrated. This would be of immense use in unambiguous identification and protection of rice hybrids, including marker-assisted production of pure hybrid rice seeds in India (Ngangkham et al. 2010; Anand et al. 2012). More recently, the efficacy of gene-based 50 K SNP chip in molecular diversity and evolutionary studies among cultivated and wild rice accessions was demonstrated (Singh et al. 2015b). The large-scale diversity analysis of Indian rice germplasm will also be useful in identifying important hot-spot (rich in trait and/or allelic diversity) rice producing regions of the country, like Eastern Indo-gangetic planes of Uttar Pradesh and Bihar for wild rice accessions as well as Eastern and North-eastern states of India for distinctive landraces (Das et al. 2013; Singh et al. 2013b, 2013c). Considering the difficulties involved in genotypic and phenotypic characterization of the huge set of available germplasm resources of rice, efforts have been made currently in India to constitute the core and mini-core collections in rice by identifying the largest amount of genetic diversity with a minimum number of accessions. In order to constitute core/minicore germplasm collections, diverse popularly grown and rarely cultivated traditional varieties and landraces belonging to *indica*, which constitute about 80 % of total cultivated Indian rice, have been assessed for phenotypic and genotypic diversity analysis (Prashanth et al. 2002). To constitute a trait-specific mini-core in rice, diversity analysis of germplasm lines have also been performed targeting various yield-component, biotic stress tolerance and grain quality traits (grain color and micronutrient content) (Banumathy et al. 2010; Singh et al. 2011a; Prasad et al. 2013; Patel et al. 2014). With the multi-institutional efforts of Indian scientists, recently a set of 98 accessions belonging to a core/mini-core collection representing 94 % allelic diversity of the total 6912 rice germplasm lines has been constituted utilizing both marker-based genotyping and phenotyping strategies and different precise statistical measures (Tiwari et al. 2015a). Similarly, a core set of 701 accessions representing 99.9 % allelic diversity of total 6984 rice germplasm lines belonging to North Eastern region (considered as hot-spot region) of India has been developed (Choudhury et al. 2014). These readily available core/mini-core germplasm resources of rice have been phenotyped for diverse agronomic traits, including yield component and abiotic/biotic stress tolerance traits at different geographical locations (multi-environment) and hot-spot regions of India for multiple years in field.

Based on phenotypic and genotypic characterization of germplasm lines, accessions contrasting for major yield component and stress tolerance traits have been selected and utilized as parents for generation of advanced bi-parental and back-cross mapping populations such as RILs (recombinant inbred lines) and NILs (near isogenic lines) in rice. One of the upland *indica* (*aus*) rice accession, Nagina 22, has been induced with EMS (ethyl methane sulfonate) mutagen to generate and characterize 22,292 mutant lines through an Indian National initiative involving several research institutes (Mohapatra et al. 2014). These mutant lines were phenotyped with a collaborative effort for a range of traits, which led to identify mutants for flowering, maturity, grain number and size, plant growth and architecture, yield, resistance to blast and bacterial leaf blight diseases, tolerance to drought, heat, salinity and herbicide and phosphorus use efficiency (Ashokkumar et al. 2013; Kulkarni et al. 2013; Poli et al. 2013; Mohapatra et al. 2014; Panigrahy et al. 2014; Lima et al. 2015). These mutant repositories could serve as a valuable resource for mining of novel functional alleles regulating qualitative and quantitative traits for genetic improvement in rice.

### Genome-Wide Scanning of Trait-Associated Functionally Relevant Molecular Tags

Investigations by Indian researchers in the context of high-throughput SSR and SNP marker-based genotyping in advanced generation bi-parental mapping population enabled to construct high-density genetic linkage and functional transcript maps and hastened identification and mapping of genes/QTLs associated with agronomic traits in rice. For instance, about 300 QTLs governing growth and grain yield contributing traits (panicle length, days to heading/flowering, plant height, grain yield, grain weight, grain size and grain length), various quality component traits (amylose content, cooked kernel elongation ratio, aroma, grain physico-chemical and cooking quality traits, and iron and zinc concentration nutritional quality traits) and stress tolerance traits (sheath blight, drought and salinity resistance) have been identified and mapped on high-density SSR and SNP marker-based genetic linkage maps derived from diverse *indica* and aromatic rice based mapping population of rice (Marri et al. 2005; Ammar et al. 2009; Amarawathi et al. 2008; Channamallikarjuna et al. 2010; Vikram et al. 2011; Salunkhe et al. 2011; Anand et al. 2012, 2013; Anuradha et al. 2012; Guleria et al. 2012; Marathi et al. 2012; Meenakshisundaram et al. 2011; Shanmugavadivel et al. 2013). The constructed high-density genetic linkage maps have been integrated with sequence-based physical map and improved the resolution and accuracy of trait-specific genes/QTLs identification. Utilizing such map-based cloning strategy, genes harbouring major QTLs associated with diverse agronomic traits, including *Pi-K*^*h*^ (*Pi54*) and PPR (pentatricopeptide repeat) genes regulating blast resistance and fertility restoration have been identified in rice (Sharma et al. 2005; Reddy et al. 2008; Ngangkham et al. 2010). Recently, the integration of QTL mapping and microarray-based genome-wide transcriptome profiling of parents and bulks of homozygous RILs has been found to be a powerful approach to narrow down the number of candidate genes underlying the QTLs of interest and for isolating the possible genes regulating the traits. Using such strategy, a candidate gene belonging to glycosyl hydrolase family regulating the number of grains per panicle in a major QTL region (*qGN4-1*) has been mapped on chromosome 4, and two genes encoding integral transmembrane protein DUF6 and cation chloride cotransporter co-localized in the significant QTL intervals on chromosomes 1, 8, and 12 for salt ion concentrations and two known genes, *badh1* and *badh2* (betaine aldehyde dehydrogenase) at QTL interval governing aroma have been identified in rice (Deshmukh et al. 2010; Pandit et al. 2010; Sharma et al. 2011; Pachauri et al. 2014).

Efforts have been made by Indian scientists to mine novel allelic variants in the known cloned genes such as *Pi54* and *Pita* regulating blast resistance, *Xa21*, *Xa26* and *xa5* for bacterial blight resistance, *OsDREB1F* for drought tolerance, *GW2* for grain size/weight and nine candidate stress responsive genes governing abiotic and biotic stress tolerance traits in rice (Singh et al. 2010c; Das et al. 2012; Parida et al. 2012; Dixit et al. 2013; Kumari et al. 2013; Devanna et al. 2014; Ramkumar et al. 2014; Bimolata et al. 2015; Singh et al. 2015c, 2015d; Thakur et al. 2015). In addition, diverse mutant populations and natural germplasm collections (core and mini-core) available for rice have been assayed through TILLING (targeting induced local lesions in genomes) and EcoTILLING to mine novel functional allelic variants in the known/candidate genes associated with various agronomic traits (Ashokkumar et al. 2013; Kulkarni et al. 2013, 2014; Poli et al. 2013; Mohapatra et al. 2014; Panigrahy et al. 2014; Lima et al. 2015). More recently, GWAS (genome-wide association study) using the genotyping information of custom designed Illumina Infinium array based on 6000 SNPs (present in many stress responsive genes distributed across 12 chromosomes with average distance of <100 kb between SNP loci) assayed in 220 accessions (association panel), has been performed to identify 20 SNPs in known/candidate genes significantly associated with Na^+^/K^+^ ratio and 44 with other salinity stress tolerance contributing traits in rice (Kumar et al. 2015).

### Marker-Assisted Breeding for Rice Crop Improvement

Many successful endeavors have been made by Indian rice molecular breeders to introgress and pyramid the superior functional genes and major QTLs/alleles regulating complex yield/quality component and stress tolerance traits into diverse rice genotypes especially using marker-assisted selection (MAS) for their genetic enhancement. The genetic improvement of Basmati rice for yield, quality and resistance to bacterial leaf blight (*Xa21*, *xa4*, *xa13*, *xa5*, *Xa33t*, *xa34t* and *Xa38*) and blast (*Pi1*, *Pi2*, *Pi5*, *Pi9*, *Pi54*, *Pib*, *Piz*, *Piz5*, *Pi-ta* and *Pi54*/*Pi-K*^*h*^), brown plant hopper [*Bph-3/17/18/20/21* and *Bph18(t)*], sheath blight (*qSHB*) and gall midge (*Gm4* and *Gm8*) diseases has been performed by pyramiding the multiple genes/QTLs through marker-assisted back-crossing (MABC)/marker-assisted foreground and background selection (Joseph et al. 2004; Sharma et al. 2005; Cheema et al. 2008; Sundaram et al. 2008, 2009; Gopalakrishnan et al. 2008; Himabindu et al. 2010; Basavaraj et al. 2010; Madhavi et al. 2011; Sama et al. 2010, 2012; Hari et al. 2011,2013; Singh et al. 2011b; Natarajkumar et al. 2012; Sujatha et al. 2010, 2013; Pandey et al. 2013; Pradhan et al. 2015). A multi-institutional National network project with an objective to introgress known cloned QTLs regulating drought (*DTY1.1*, *DTY2.1*, *DTY2.2*, *DTY3.1*, *DTY3.2*, *DTY9.1* and *DTY12.1*), flood (sub-mergence) (*Sub1*) and salinity stress (*Saltol*) tolerance (http://india.irri.org/mega-projects-in-india, Singh et al. 2015b) into high-yielding mega rice varieties (ADT46, Bahadur, MTU1075, Pooja, Rajendra, Mahsuri, Ranjit, ADT39, Pusa44, ADT45, Gayatri and Savitri) of India through MAS for their genetic enhancement for target traits is under progress. This eventually may lead to development of certain diverse genetically-tailored high-yielding and climate resilient early maturing Indian rice varieties for sustaining food security.

## Conclusions

The rice genome sequence has served as a catalyst to accelerate efforts on the functional analysis of genes/QTLs by reverse and forward genetics in India. This is coupled with use of genomics-assisted breeding of rice to improve traits such as yield, abiotic stress tolerance and biotic stress resistance. Diverse varieties of rice are grown in different regions of India and concerted efforts are required to introgress genes/QTLs for desirable traits into them. Some of these efforts have already led to release of improved varieties for submergence tolerance and biotic stress resistance. Indian rice researchers have highly benefited from new knowledge generated worldwide and from their own collaborative efforts in crop improvement programs. It is hoped that appropriate regulatory process will help move transgenic rice also to the field level evaluation and deregulation in due course. In the meantime, several projects related to molecular dissection of desirable agronomic traits of rice and introgression of appropriate genes/QTLs have been initiated. India is also poised to contribute to international projects in the area of functional genomics of rice and looks forward to launch of activities like riceENCODE. It is hoped that the outcome of rice genomics would go a long way to influence research in other related crops as well.
